# Supplementary material for: Insect decline in forests depends on species’ traits and may be mitigated by management
Source: Commun Biol. 2023 Apr 4;6:338. doi: 10.1038/s42003-023-04690-9 (PMC10073207; doi:10.1038/s42003-023-04690-9)
Supplement: Supplementary file 2 — Supplementary Information [file 42003_2023_4690_MOESM2_ESM.pdf]

Supplementary Information

Insect decline in forests depends on species' traits and can be mitigated by management

Michael Staab, Martin M. Gossner, Nadja K. Simons, Rafael Achury, Didem Ambarlı, Soyeon Bae, Peter Schall, Wolfgang W. Weisser, and Nico Blüthgen

To whom correspondence may be addressed. Email: michael.staab1@tu-darmstadt.de

Contents

Supplementary Methods……………………...…………………..…2

Supplementary Figure 1. Map of study regions……………………………………………….……5

Supplementary Figure 2. Site-level correlations based on raw vs. log-transformed data……....6

Supplementary Figure 3. Comparison of correlations between sampling regimes……………….7

Supplementary Figure 4. Correlation matrix of explanatory variables……………………………..8

Supplementary Figure 5. Comparison of analytical approaches…………………………………..9

Supplementary Figure 6. Compositional species overlap between sampling regimes………...10

Supplementary Figure 7. Relationships for site-level correlations of herbivores….…………....11

Supplementary Figure 8. Relationships for site-level correlations of myceto-detritivores…....12

Supplementary Figure 9. Relationships for site-level correlations of omnivores….………........13

Supplementary Figure 10. Relationship for site-level correlations of carnivores….……...…….15

Supplementary Figure 11. Comparative matrix of all site-level correlations……………......…....16

Supplementary Figure 12. Distribution of species-level correlations……………..…..……..….…17

Supplementary Figure 13. Species-level correlations and yearly occurrence……………………18

Supplementary Table 1. Overview of explanatory variables…………………...….…..…………….19

Supplementary Table 2. Details on site-level correlations……….………….…………..………...20

Supplementary Table 3. Statistical details for species-level analyses……………………...……21

Supplementary Table 4. Pairwise contrast among trophic groups in species-level analyses…22

References cited in Supplementary Information……………………………………….…...…..…23

Supplementary Methods

Flight interception traps consisted of two crossed transparent plastic panels (40 cm x 60 cm) acting as flight barriers. At the top and bottom (Knuff et al. 2019), funnels connected to collection jars filled with 3% $CuSO_4$ solution and a drop of detergent to break surface tension. Traps were placed at three randomly selected site corners at approximately 1.5 m above the ground and operated from March to October (six sampling rounds, yearly-sampled intensive sites) or from March to July (four sampling rounds, remaining sites), with collection jars replaced in approximately monthly intervals. Two of the three traps per plot and sampling round were randomly selected for further processing. In order to harmonize sampling effort among sites that are sampled yearly vs. triennially, site-level analyses were based on three sampling rounds covering May, June and July, the main flight-activity period of insects in Central Europe (Shi et al. 2021). Catches from the first sampling round (March-April; all sites) were excluded as this period could for the triennially-sampled sites not be sampled in all years. Likewise, the last two sampling rounds (August-October) were excluded, as these are only available for the yearly-sampled sites.

To characterize the forest at each 100 m x 100 m (1 ha) site, we inventoried living trees, stumps and deadwood, and applied terrestrial and airborne laser scanning. At each site, forest inventories were conducted in two separate periods, 2009-2011 and 2015-2016, with usually six years between inventories at each site. All living trees > 7 cm diameter at breast height were mapped, identified and measured. For deadwood, items > 25 cm were recorded at the whole 1 ha site. Deadwood items with a diameter between 7-25 cm were recorded on two line transects (2 m wide) along the site diagonals (141.42 m each) and extrapolated to 1 ha. More details on tree and deadwood inventories are reported in Schall et al. (2018). From these comprehensive inventory data we derived *harvesting intensity*, the basal area of timber harvested in the ~10–15 year period (assessed from decay stage of stumps) before the start of insect sampling divided by the total basal area of living trees and harvested timber (following Kahl & Bauhus 2014). To express the *change in harvesting*, we used the basal area of trees felled from 2008 to 2017 (i.e. insect sampling period) (Schall et al. 2018). *Deadwood volume* was calculated as the volumetric sum of all stumps, laying and standing deadwood items >7 cm diameter. The *proportion of non-native trees* was expressed as the volume of living trees, harvested trees and deadwood that do not belong to the native tree species composition (mainly spruce and pine) at the sites divided by the total volume of all living trees, harvested trees and deadwood. For characterizing the conditions at the beginning of the insect time series, we use data from the first forest inventory. In turn, for describing changes in forest

properties over time we calculated for deadwood volume and non-native trees the change between the second and first inventory ($condition_{second\ inventory} – condition_{first\ inventory}$). Thus, positive values of change indicate a higher value of the respective forest property in the second inventory (e.g. an increase in deadwood volume over time).

The vertical structure of each site, a proxy for vertical heterogeneity, was characterized with terrestrial laser scanning (LiDAR) in summer 2014 and again in summer 2019. Per 1 ha site, nine evenly-distributed scans were conducted. From the original point clouds (density of 44.4 million beams per scan) the *effective number of layers* (ENL) was calculated as the inverse Simpson index of filling of 1 m horizontal layers (Ehbrecht et al. 2016). Higher ENL values thus indicate vertically more evenly layered vegetation (i.e. multi-layered forest). From the exactly same laser scans, we calculated *canopy openness* as the percentage of sky pixels of a simulated hemispherical image for an opening angle of 60° (Zheng et al. 2013). As for forest properties, we considered the conditions from the first scan and the change between the second and the first scan.

The landscape in a 1000 m radius around the center of each site was assessed from a range of satellite data. *Forest cover*, a surrogate of habitat availability at the landscape scale, was measured from vectorized ATKIs Basis DLM land cover data recorded in 2009 (Seibold et al. 2019). To quantify changes in forests at the landscape scale during the period covered by the insect time series, we calculated *disturbance intensity*, the percentage of forest in a 1000 m radius in which the canopy changed from 2008 to 2017 (Senf & Seidl 2021). This variable is based on continuous Landsat imagery (30 m x 30 m resolution) and includes canopy alterations due to natural disturbances (e.g. windthrow) and forestry interventions (e.g. large timber extraction). We furthermore utilized satellite-borne radar data (Sentinel-1) to assess the *heterogeneity of forests* around each site (1000 m radius). Backscatter intensities of the C-band (vertically transmitted, vertically received pulses and vertically transmitted, horizontally received pulses) for the study regions (pixel spacing 10 m) recorded in 2016 were downloaded from the ESA Scientific Hub and processed as described in Bae et al. (2019). Among the variables used in Bae et al. (2019), we selected variables representing structural heterogeneity, i.e. standard deviation and textural variables, and they were calculated for a 1000 m radius. To reduce the complexity of the data, we performed a principal component analysis (PCA) of the 30 radar-derived variables. The first two PCs accounted for 76.7% of the total variation (PC1: 53.9%, PC2: 22.8%). This approach has been shown before to representatively capture forest structure and composition (Bae et al. 2019). Only PC1 was used in analyses as PC2 imposed inflated variances in linear mixed-effects

models. Data utilizing Sentinel-1 spacecrafts are only available from 2016 onwards, precluding the assessment of change in conditions among years with insect monitoring.

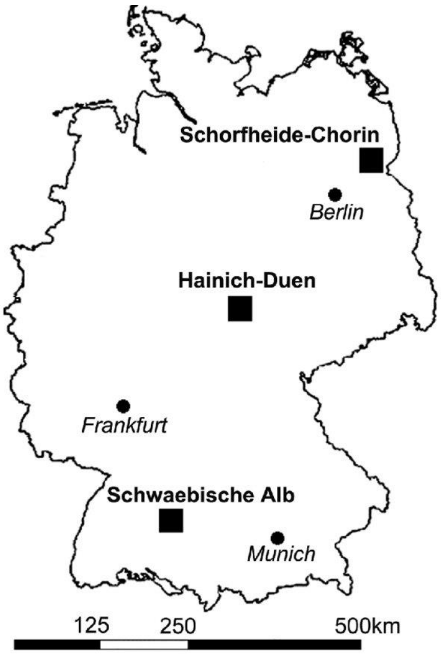

Supplementary Figure 1. Map of Germany showing the geographic location of the three Exploratories in the southwest (Schwäbische Alb), the center (Hainich-Dün) and the northeast (Schorfheide-Chorin).

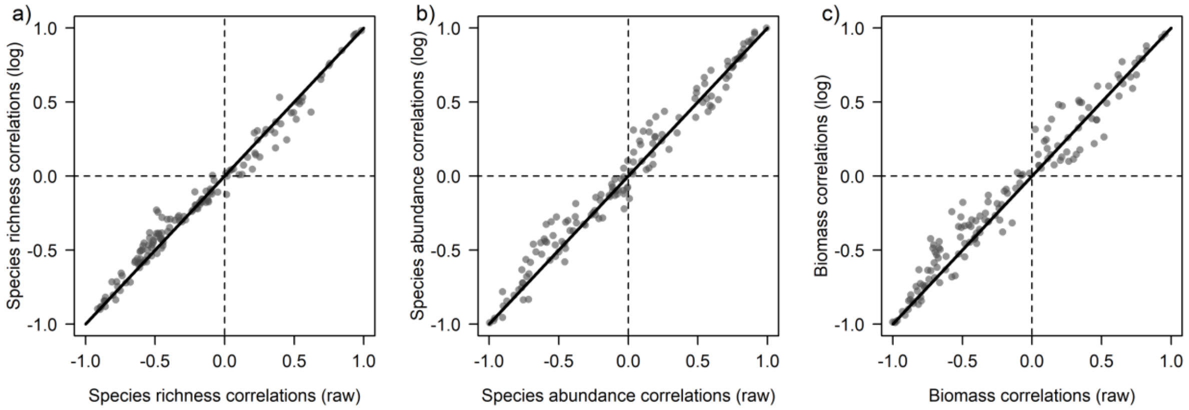

Supplementary Figure 2. Site-level correlations are invariant to whether correlations (i.e. Pearson's *r* between year and the respective community response) are calculated on raw or log-transformed data. For a) species richness (r = 0.990), b) abundance (r = 0.986) and c) biomass (r = 0.978) raw and log-transformed correlations align along the expected diagonal (solid line). Dashed horizontal and vertical lines mark null with negative values indicating sites with declining and positive values sites with increasing community responses over time.

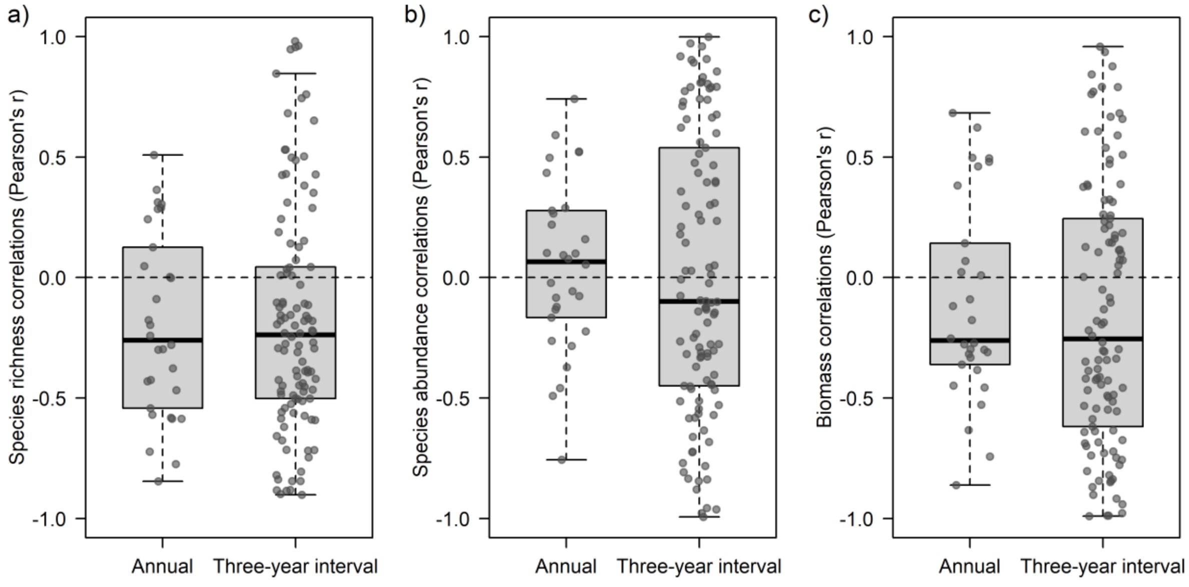

Supplementary Figure 3. Correlations per site (Pearson's *r* between year and the respective community response) for species richness a), abundance b) and biomass c) are not influenced by the sampling regime (center lines in boxplots specify the median, boxes cover the range between the lower and upper quartile, whiskers extend to 1.5x interquartile range). Annually-sampled sites (n = 30) are representative of sites sampled in three-year intervals (n = 110) and differences between sampling regimes (linear mixed-effects models, fixed effect: sampling regime, random effect: region) are not significant. Dashed horizontal lines mark null with negative values indicating sites with declining and positive values sites with increasing community responses over time.

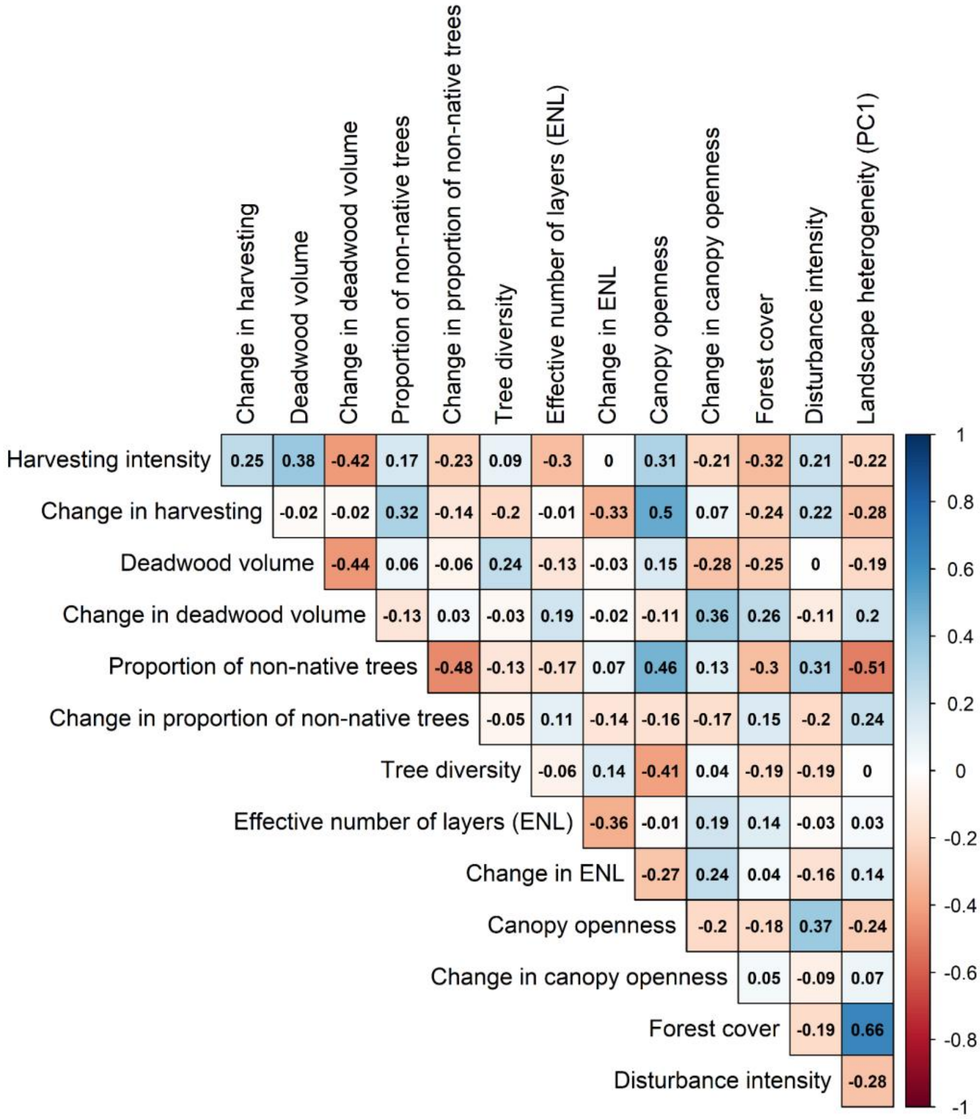

Supplementary Figure 4. Correlations (Spearman's ρ) among the explanatory variables used for site-level analyses. For explanations of variables see Supplementary Table 1.

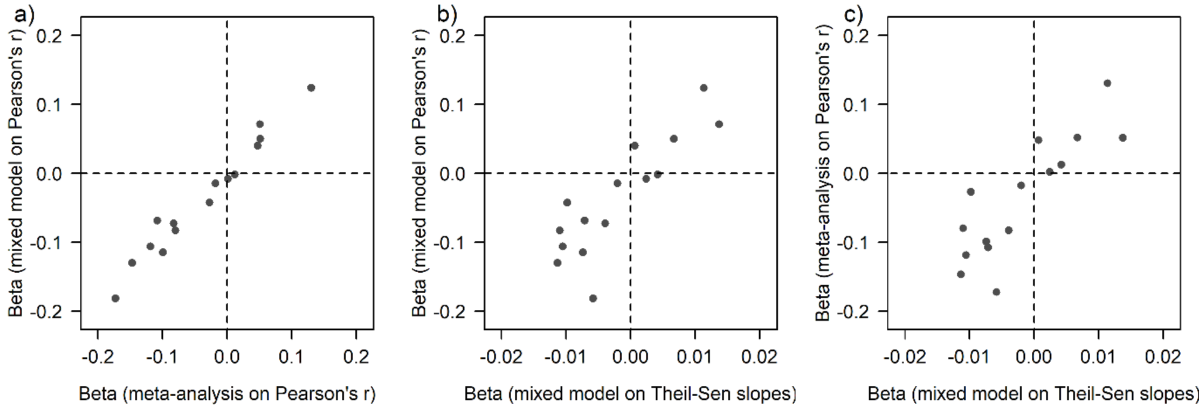

Supplementary Figure 5. Different approaches to quantify the relationships between total insect species richness and sampling year at the site-level yield highly congruent outcomes. Points indicate the respective beta coefficients of site- and landscape-level explanatory variables among analytical approaches and are correlated. a) a linear mixed-effects model with Pearson's *r* (between year and the number of species per site) as response variable (i.e. the approach used in the study) compared to a multi-level mixed effects meta-analysis with *r* as effect size, environmental variables as moderators and sampling effort as weights (r = 0.984); b) a linear mixed-effects model with Pearson's *r* as response variable compared to a linear mixed-effects model with Theil-Sen slopes (of year and the number of species per site) as response variable (r = 0.860); c) a multi-level mixed effects meta-analysis with *r* as effect size, environmental variables as moderators and sampling effort as weights compared to a linear mixed-effects model with Theil-Sen slopes as response variable (r = 0.848). Theil-Sen slopes are the median slopes of all possible pairwise slopes and were calculated with the R-package 'robslopes' (Raymaekers 2022). The meta-analysis was conducted in the R-package 'metafor' (Viechtbauer 2010). Dashed horizontal and vertical lines mark null. The high congruence among the different analytical approaches, especially in a), affirms our confidence in the suitability of using Pearson's *r* between year and the respective community response as response variable in mixed-effects models for our statistical analyses.

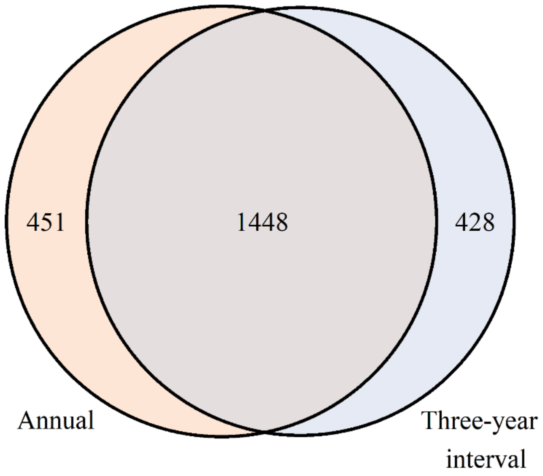

Supplementary Figure 6. Venn diagram illustrating the large compositional overlap between the 30 annually sampled and the 140 triennially sampled sites in species per region combinations (excluding singletons) available for species-level analysis. Species not shared among sampling regimes were rare. The 451 species per region combinations not contained in the annual data account for 2.1% of specimens in this data; in turn, the 428 species per region combinations not contained in the triennial data account for 2.0 % of specimens.

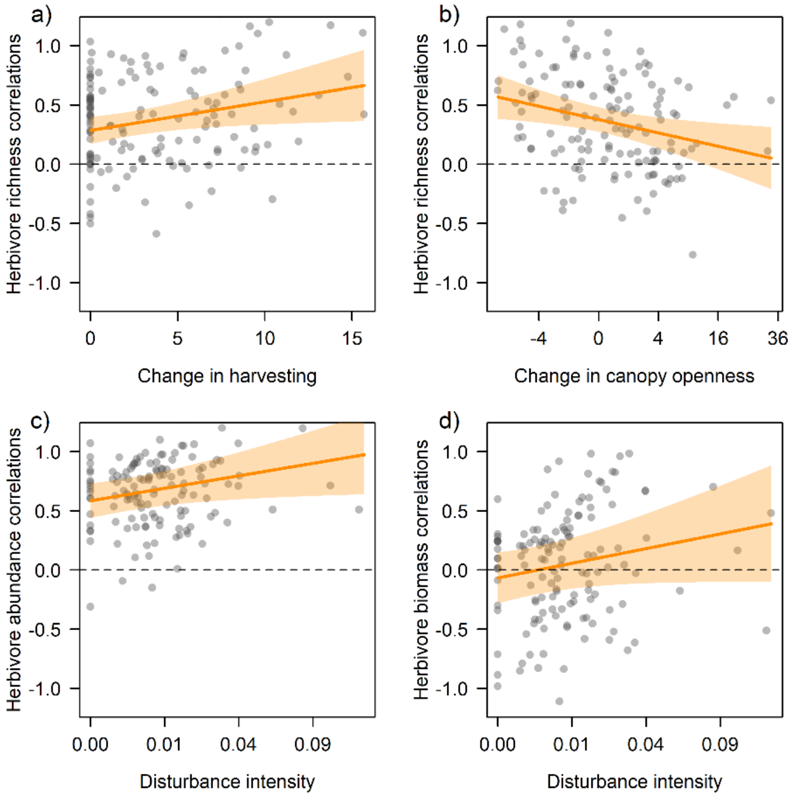

Supplementary Figure 7. Results for site-level correlations of herbivores and further environmental variables. Site-level correlations (each shown as partial residuals of Pearson's *r* between year and the respective community response) for herbivore species richness were related to a) change in harvesting and b) change in canopy openness. For herbivore abundance and biomass, correlations were related to disturbance intensity (c, d). For explanations of variables see Supplementary Table 1. Full statistical details are available in Supplementary Data 1. Regression lines (95% CI in shaded polygons) indicate the marginal predictions of linear mixed-effects models. Dashed horizontal lines mark null with negative values indicating sites with declining and positive values sites with increasing community responses over time. Note that the x-axis in b) is on a symmetric square-root scale and that the x-axes in c) and d) are on a square-root scale.

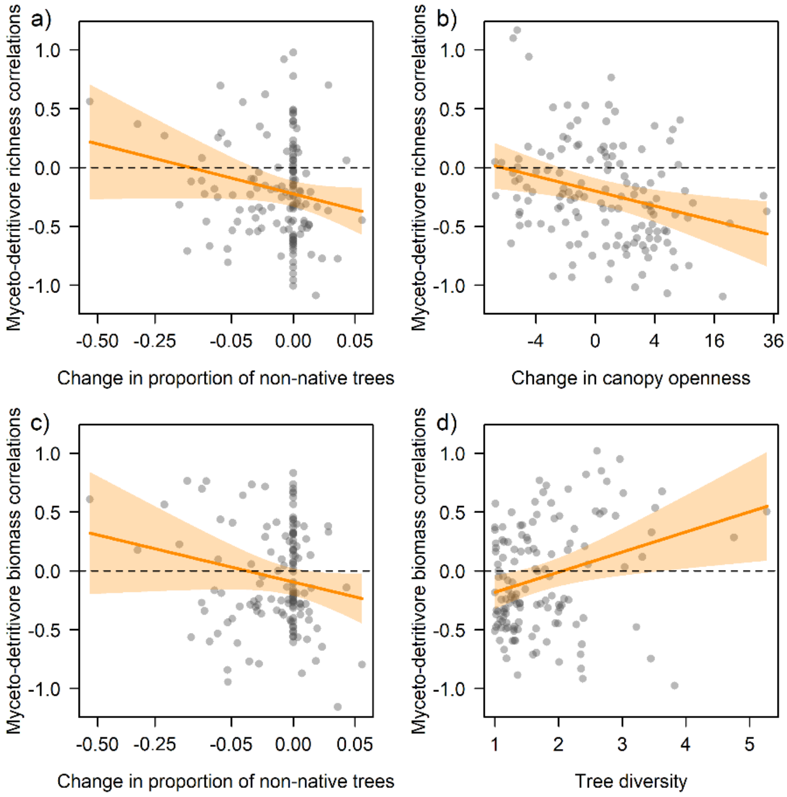

Supplementary Figure 8. Results for site-level correlations of myceto-detritivores and further environmental variables. Site-level correlations (each shown as partial residuals of Pearson's *r* between year and the respective community response) for myceto-detritivore species richness were related to a) change in the proportion of non-native trees and b) change in canopy openness. For myceto-detritivore biomass, correlations were related to c) change in the proportion of non-native trees and d) tree diversity. For explanations of variables see Supplementary Table 1. Full statistical details are available in Supplementary Data 1. Regression lines (95% CI in shaded polygons) indicate the marginal predictions of linear mixed-effects models. Dashed horizontal lines mark null with negative values indicating sites with declining and positive values sites with increasing community responses over time. Note that the x-axes in a), b) and c) are on a symmetric square-root scale.

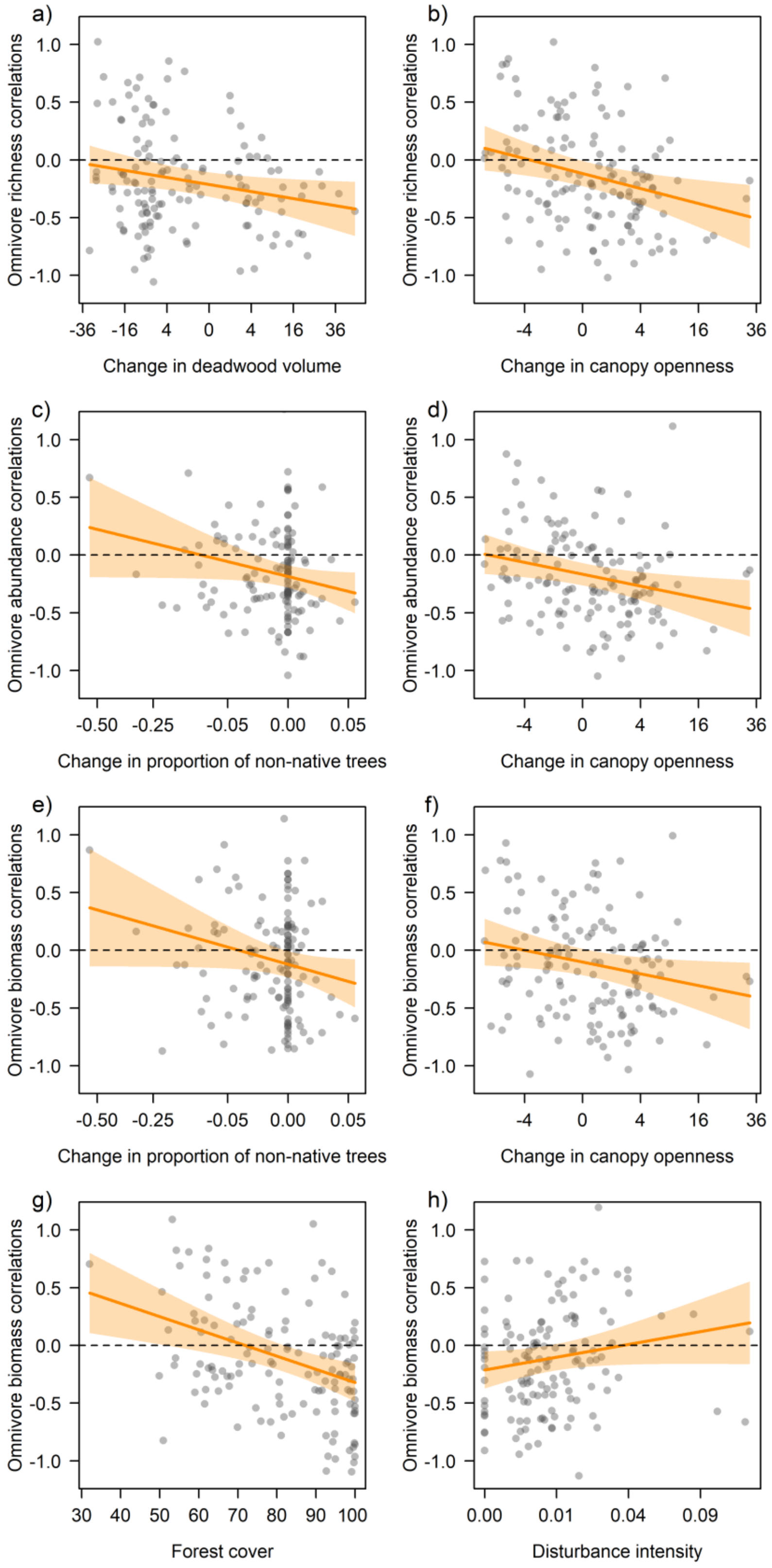

Supplementary Figure 9. Results for site-level correlations of omnivores and further environmental variables. Site-level correlations (each shown as partial residuals of Pearson's *r* between year and the respective community response) for omnivore species richness were related to a) change in deadwood volume and b) change in canopy openness. For omnivore abundance, correlations were related to c) change in the proportion of non-native trees and d)

change in canopy openness. Omnivore biomass correlations were related to e) change in the proportion of non-native trees, f) change in canopy openness, g) forest cover and h) disturbance intensity. For explanations of variables see Supplementary Table 1. Full statistical details are available in Supplementary Data 1. Regression lines (95% CI in shaded polygons) indicate the marginal predictions of linear mixed-effects models. Dashed horizontal lines mark null with negative values indicating sites with declining and positive values sites with increasing community responses over time. Note that the x-axes in a), b), c), d), e) and f) are on a symmetric square-root scale and that the x-axis in h) is on a square-root scale.

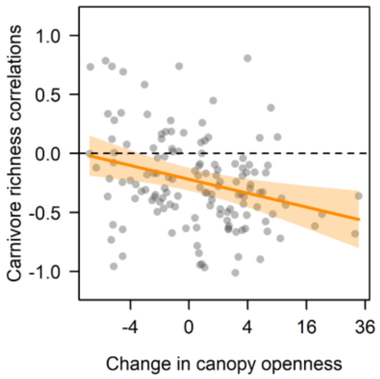

Supplementary Figure 10. Results for site-level correlations of carnivores and further environmental variables. Site-level correlations (shown as partial residuals of Pearson's *r* between year and carnivore species richness) for species richness were related to change in canopy openness. For explanations of variables see Supplementary Table 1. Full statistical details are available in Supplementary Data 1. Regression line (95% CI in shaded polygon) indicates the marginal prediction of a linear mixed-effects model. Dashed horizontal line marks null with negative values indicating sites with declining and positive values sites with increasing carnivore species richness over time. Note that the x-axis is on a symmetric square-root scale.

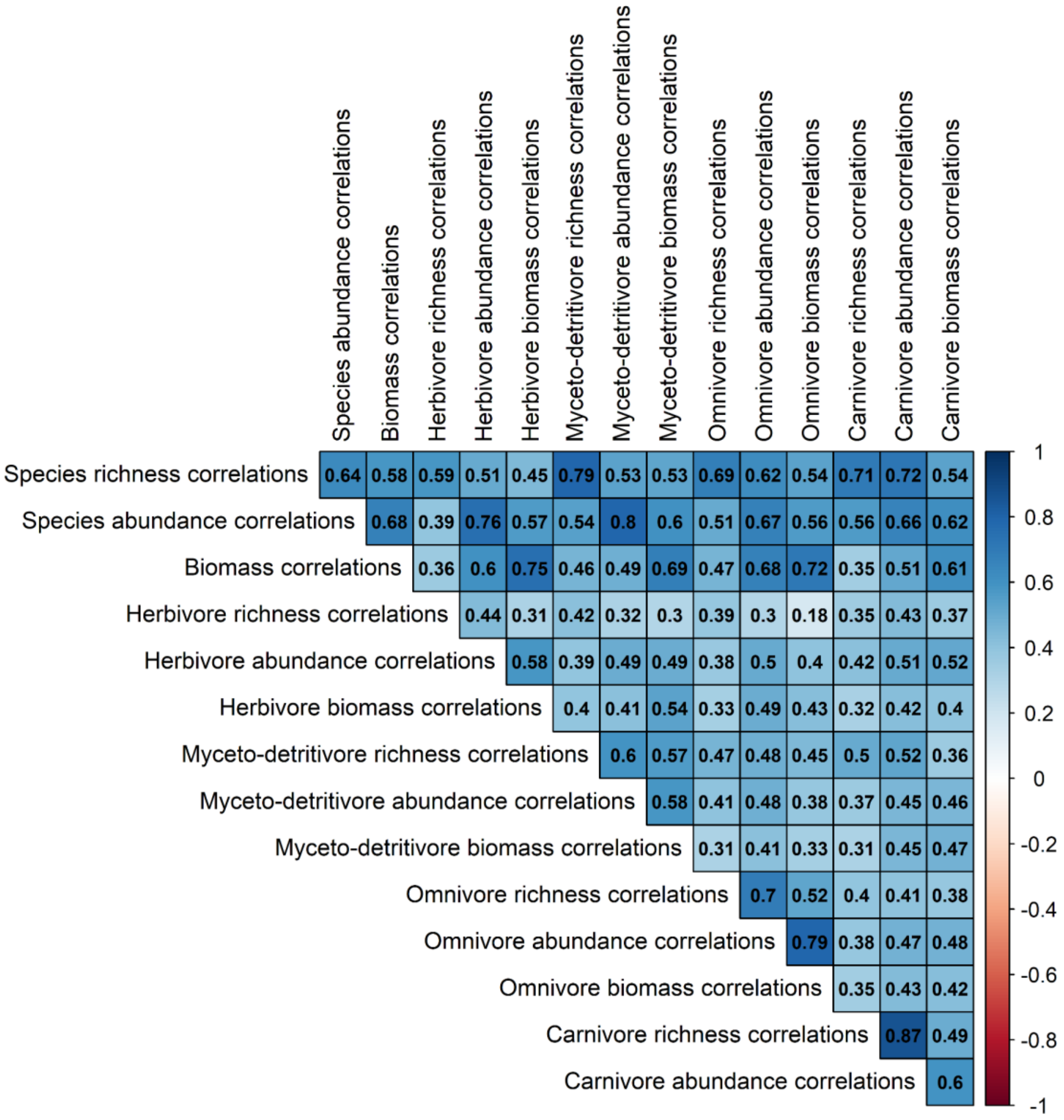

Supplementary Figure 11. Site-level correlations (expressed as Pearson's *r* between year and the respective community response) of species richness, abundance and biomass among the total insect community and all trophic groups are always positively related. All pairwise correlations (Spearman's ρ) are significant (at $p < 0.05$).

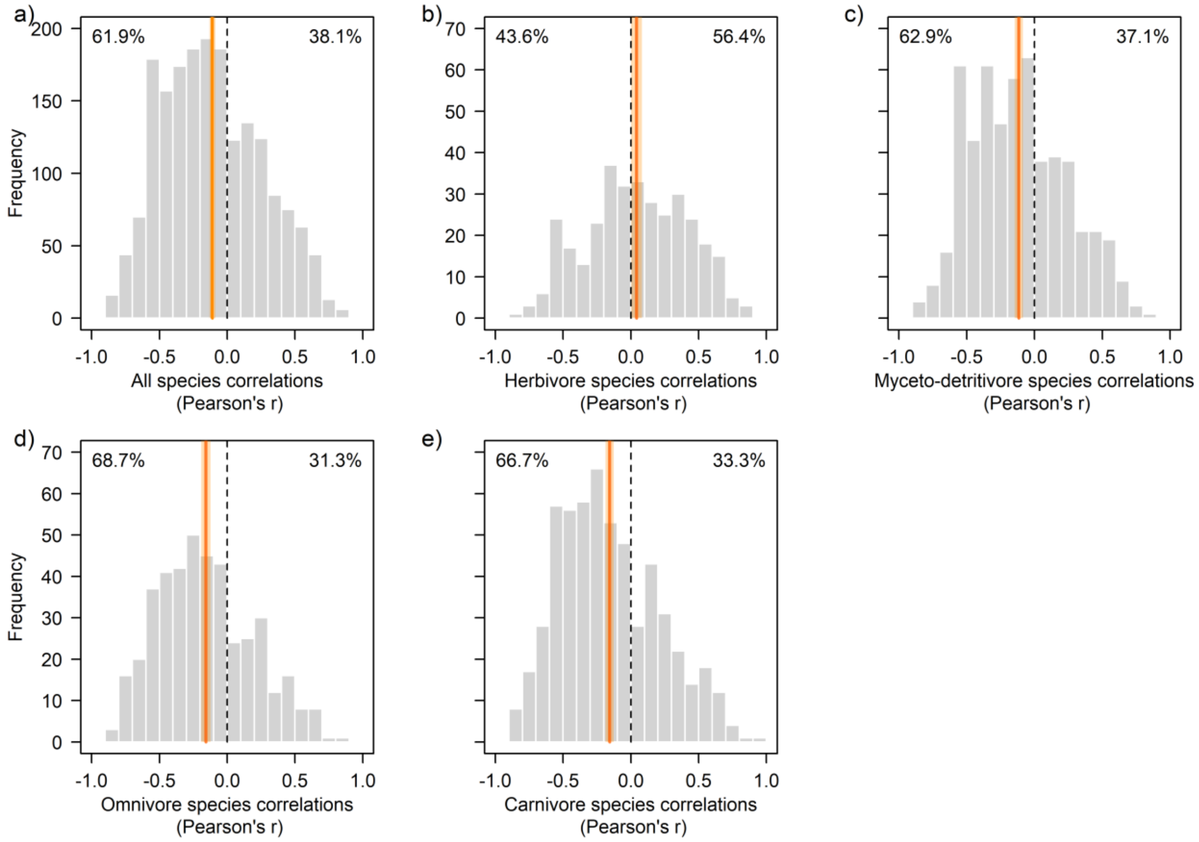

Supplementary Figure 12. Distribution of species-level correlations (Pearson's *r* between year and the number of individuals per species in a region, excluding single occurrences) for a) all species, b) herbivores, c) myceto-detritivores, d) omnivores, and e) carnivores. Dashed vertical lines mark null with negative values indicating species with declining and positive values species with increasing individual numbers over time. With the exception of herbivores, all average correlations are negative (bold vertical line: mean, shaded polygons: 95% CI). Percentages in each panel give the proportion of species with negative (left) and positive (right) correlations.

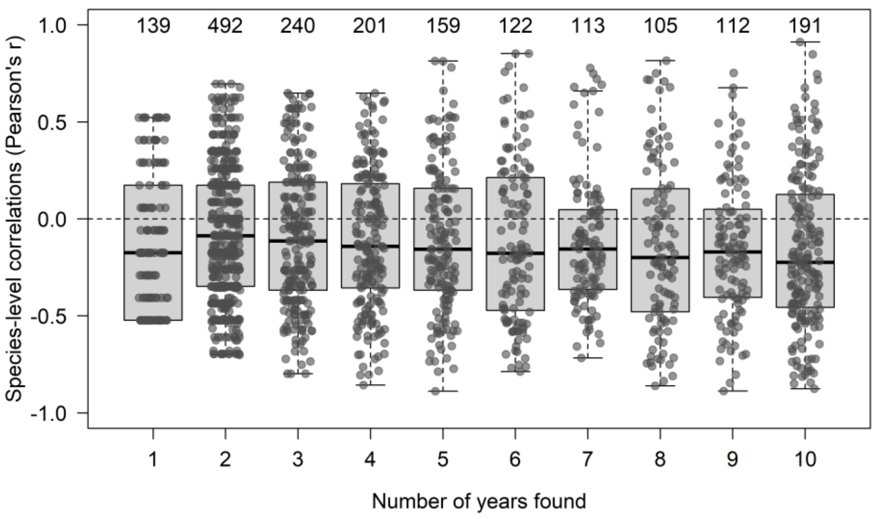

Supplementary Figure 13. Species-level correlations (i.e. Pearson's *r* between year and the number of individuals per species in a region) are independent of the number of years a species was collected per region (center lines in boxplots specify the median, boxes cover the range between the lower and upper quartile, whiskers extend to 1.5x interquartile range). Numbers above boxes indicate the number of species*region combinations per number of years. Dashed horizontal lines mark null with negative values indicating species with declining and positive values species with increasing individual numbers over time.

Supplementary Table 1. Overview of explanatory variables used to describe forest conditions at the site (100 m x 100 m) and landscape scale (1000 m radius).

Variable	Definition	Method	Source
Site scale			
Harvesting intensity	Basal area of timber harvested in the ~10–15-year period before the start of insect sampling divided by the total basal area of living trees and harvested timber (each considering trees > 7 cm diameter)	Forest inventory	Kahl & Bauhus 2014
Change in harvesting	Basal area of timber harvested between 2008 and 2017 (insect sampling period)	Forest inventory	Schall et al. 2018
Deadwood volume	Volumetric sum of all stumps, laying and standing deadwood (> 7 cm diameter)	Forest inventory	Kahl & Bauhus 2014
Change in deadwood volume	Deadwood volume in the 2nd inventory minus 1st inventory	Forest inventory	Kahl & Bauhus 2014
Proportion of non-native trees	Proportion of tree basal area not native at a site divided by the basal area of all trees (> 7 cm diameter)	Forest inventory	Kahl & Bauhus 2014
Change in proportion of non-native trees	Proportion of non-native trees in the 2nd inventory minus 1st inventory	Forest inventory	Kahl & Bauhus 2014
Tree diversity	Exponential Shannon index (*e^H*) based on the basal area of trees (> 7 cm diameter)	Forest inventory	Schall et al. 2018
Effective number of layers (ENL)	Inverse Simpson index of filling of 1 m horizontal layers (9 scans per site)	Terrestrial laser scanning	Ehbrecht et al. 2016
Change in effective number of layers	ENL 2nd scan minus ENL 1st scan	Terrestrial laser scanning	Ehbrecht et al. 2016
Canopy openness	Percentage of sky pixels in a hemispherical image (60° opening angle)	Terrestrial laser scanning	Zheng et al. 2013
Change in canopy openness	Canopy openness 2nd scan minus canopy openness 1st scan	Terrestrial laser scanning	Zheng et al. 2013
Landscape scale			
Forest cover	Percentage of area (1000 m radius) around each site covered by forest	ATKIs DLM land cover data	Seibold et al. 2019
Disturbance intensity	Percentage of forest area (1000 m radius, 30 x 30 m resolution) affected by disturbance (change in canopy) from 2008 – 2017	Landsat	Senf & Seidl 2021
Landscape heterogeneity (PC1)	1st principal component of radar intensities (pixel spacing of 10 m, 1000 m radius) representing structural heterogeneity	Radar satellite (Sentinel-1)	Bae et al. 2019

Supplementary Table 2. Site-level correlations in species richness, abundance and biomass. Shown are the mean and 95% CI of correlations (Pearson's *r* between year and the respective community response), number of species and individuals for the total insect community and each trophic group.

	Mean	95% CI	Species	Individuals
Total community			1,805	120,996
Richness correlations	-0.182	-0.257 – -0.106		
Abundance correlations	0.011	-0.079 – 0.100		
Biomass correlations	-0.152	-0.237 – -0.066		
Herbivores			361	38,100
Richness correlations	0.306	0.232 – 0.379		
Abundance correlations	0.389	0.304 – 0.468		
Biomass correlations	-0.023	-0.112 – 0.067		
Myceto-detritivores			443	38,531
Richness correlations	-0.272	-0.347 – 0.197		
Abundance correlations	-0.137	-0.217 – -0.057		
Biomass correlations	-0.171	-0.254 – -0.088		
Omnivores			418	28,970
Richness correlations	-0.207	-0.288 – -0.125		
Abundance correlations	-0.237	-0.310 – -0.164		
Biomass correlations	-0.165	-0.248 – -0.082		
Carnivores			583	15,395
Richness correlations	-0.255	-0.326 – -0.184		
Abundance correlations	-0.299	-0.371 – -0.223		
Biomass correlations	-0.117	-0.214 – -0.020		

Supplementary Table 3. Results of the linear mixed-effects models testing the relationship between species-level correlations (Pearson's *r* between year and the number of individuals per species in a region) and species' traits for all species, persistent species (found in 6 or more years per region) and unsteady species (found in 5 or fewer years per region). Shown are *F*-values of type II tests with p-values based on Kenward-Roger-approximated degrees of freedom. Estimates (± SE) for continuous variables are given in parentheses. Significant relationships ($p < 0.05$) are printed in bold.

	F-value (Estimate ± SE)	p-value
All species		
**Body length**	**5.805 (-0.026 ± 0.011)**	**0.016**
**Incidence**	**17.668 (-0.039 ± 0.009)**	**<0.001**
**Trophic group**	**9.764**	**<0.001**
Dispersal ability	0.664	0.617
Stratum use	2.425	0.064
Persistent species		
Body length	0.868 (-0.017 ± 0.018)	0.352
Incidence	2.979 (-0.027 ± 0.016)	0.084
**Trophic group**	**4.453**	**0.004**
Dispersal ability	1.082	0.363
Stratum use	2.194	0.087
Unsteady species		
**Body length**	**8.259 (-0.033 ± 0.011)**	**0.004**
**Incidence**	**8.764 (-0.029 ± 0.010)**	**0.003**
**Trophic group**	**6.512**	**<0.001**
Dispersal ability	0.700	0.592
Stratum use	1.162	0.323

Supplementary Table 4. Pairwise contrasts for species-level correlations (Pearson's *r* between year and the number of individuals per species in a region) among trophic groups for all species, persistent species (found in 6 or more years per region) and unsteady species (found in 5 or fewer years per region). P-values are Bonferroni-Holm-corrected for multiple comparisons and based on Kenward-Roger-approximated degrees of freedom. Significant contrasts ($p < 0.05$) are printed in bold.

Contrast	Estimate ± SE	*t*-ratio	p-value
All species			
**Herbivore – Myceto-detritivore**	**0.140 ± 0.037**	**3.820**	**0.001**
**Herbivore – Omnivore**	**0.176 ± 0.037**	**4.783**	**<0.001**
**Herbivore – Carnivore**	**0.183 ± 0.036**	**5.088**	**<0.001**
Myceto-detritivore – Omnivore	0.037 ± 0.029	1.255	0.419
Myceto-detritivore – Carnivore	0.044 ± 0.028	1.590	0.336
Omnivore – Carnivore	0.007 ± 0.029	0.250	0.803
Persistent species			
**Herbivore – Myceto-detritivore**	**0.181 ± 0.059**	**3.077**	**0.009**
**Herbivore – Omnivore**	**0.201 ± 0.059**	**3.397**	**0.004**
**Herbivore – Carnivore**	**0.185 ± 0.059**	**3.123**	**0.009**
Myceto-detritivore – Omnivore	0.020 ± 0.048	0.418	1.000
Myceto-detritivore – Carnivore	0.004 ± 0.047	0.085	1.000
Omnivore – Carnivore	-0.016 ± 0.049	-0.324	1.000
Unsteady species			
**Herbivore – Myceto-detritivore**	**0.105 ± 0.041**	**2.563**	**0.042**
**Herbivore – Omnivore**	**0.151 ± 0.042**	**3.624**	**0.002**
**Herbivore – Carnivore**	**0.165 ± 0.040**	**4.150**	**<0.001**
Myceto-detritivore – Omnivore	0.046 ± 0.032	1.455	0.292
Myceto-detritivore – Carnivore	0.060 ± 0.029	2.084	0.112
Omnivore – Carnivore	-0.015 ± 0.031	-0.475	0.635

References cited in Supplementary Information

Bae S, Levick SR, Heidrich L, et al. (2019) Radar vision in the mapping of forest biodiversity from space. *Nat. Commun.* 10: 4757. https://doi.org/10.1038/s41467-019-12737-x

Ehbrecht M, Schall P, Juchheim J, et al. (2016) Effective number of layers: A new measure for quantifying three-dimensional stand structure based on sampling with terrestrial LiDAR. *For. Ecol. Manage.* 380: 212–223. https://doi.org/10.1016/j.foreco.2016.09.003

Kahl T, Bauhus J (2014) An index of forest management intensity based on assessment of harvested tree volume, tree species composition and dead wood origin. *Nat. Conserv.* 7: 15–27. https://doi.org/10.3897/natureconservation.7.7281

Knuff AK, Winiger N, Klein AM, et al. (2019) Optimizing sampling of flying insects using a modified window trap. *Methods Ecol. Evol.* 10: 1820–1825. https://doi.org/10.1111/2041-210x.13258

Raymaekers J (2022) *robslopes: Fast Algorithms for Robust Slopes*. R package version 1.1.1. https://CRAN.R-project.org/package=robslopes

Schall P, Schulze E-D, Fischer M, et al. (2018). Relations between forest management, stand structure and productivity across different types of Central European forests. *Basic Appl. Ecol.* 32: 39–52. https://doi.org/10.1016/j.baae.2018.02.007

Seibold S, Gossner MM, Simons NK, et al. (2019) Arthropod decline in grasslands and forests is associated with landscape-level drivers. *Nature* 574: 671–674. https://doi.org/10.1038/s41586-019-1684-3

Senf C, Seidl R (2021) Mapping the forest disturbance regimes of Europe. *Nat. Sustain.* 4: 63–70. https://doi.org/10.1038/s41893-020-00609-y

Shi X, Schmid B, Tschanz P, et al. (2021) Seasonal trends in movement patterns of birds and insects aloft simultaneously recorded by radar. *Remote Sens.* 13: 1839. https://doi.org/10.3390/rs13091839

Viechtbauer W (2010) Conducting meta-analyses in R with the metafor package. *J. Stat. Soft.* 36: 1–48. https://doi.org/10.18637/jss.v036.i03

Zheng G, Moskal LM, Kim SH (2013) Retrieval of effective leaf area index in heterogeneous forests with terrestrial laser scanning. *IEEE Trans. Geosci. Remote. Sens.* 51: 777–786. https://doi.org/10.1109/TGRS.2012.2205003
