# Supplementary material for: Insect decline in forests depends on species’ traits and may be mitigated by management
Source: Commun Biol. 2023 Apr 4;6:338. doi: 10.1038/s42003-023-04690-9 (PMC10073207; doi:10.1038/s42003-023-04690-9)
Supplement: Supplementary file 3 — Description of Additional Supplementary Files [file 42003_2023_4690_MOESM3_ESM.pdf]

Description of Additional Supplementary Files

File Name: Supplementary Data 1

Description: Results of linear mixed-effects models testing the relationships between site-level correlations with sampling year (Pearson's r) of species richness, richness accounted for abundance, abundance and biomass and explanatory variables at the site and landscape scale. Significant relationships ($p < 0.05$) are printed in bold. For explanations of variables see Supplementary Table 1. P-values are calculated based on Kenward-Roger-approximated degrees of freedom.

File Name: Supplementary Data 2

Description: Results of linear mixed-effects models testing the relationships between site-level correlations with sampling year (Pearson's r) of species richness, richness accounted for abundance, abundance and biomass and explanatory variables at the site and landscape scale. Significant relationships ($p < 0.05$) are printed in bold. For explanations of variables see Supplementary Table 1. P-values are calculated based on Kenward-Roger-approximated degrees of freedom. Analyses reported here are identical to those in Supplementary Data 1 but include the respective starting conditions in the first sampling year 2008 as covariate.
