# Supplementary material for: Insect decline in forests depends on species’ traits and may be mitigated by management
Source: Commun Biol. 2023 Apr 4;6:338. doi: 10.1038/s42003-023-04690-9 (PMC10073207; doi:10.1038/s42003-023-04690-9)
Supplement: Supplementary file 4 — Supplementary Data 1 [file 42003_2023_4690_MOESM4_ESM.pdf]

Supplementary Data 1

Insect decline in forests depends on species' traits and can be mitigated by management

Michael Staab, Martin M. Gossner, Nadja K. Simons, Rafael Achury, Didem Ambarlı, Soyeon Bae, Peter Schall, Wolfgang W. Weisser, and Nico Blüthgen

To whom correspondence may be addressed. Email: michael.staab1@tu-darmstadt.de

Results of linear mixed-effects models testing the relationships between site-level correlations with sampling year (Pearson's *r*) of species richness, richness accounted for abundance, abundance and biomass and explanatory variables at the site and landscape scale. Significant relationships ($p < 0.05$) are printed in bold. For explanations of variables see Supplementary Table 1. P-values are calculated based on Kenward-Roger-approximated degrees of freedom.

	Estimate ± SE	*t*-value	p-value
**Total insect community**			
Richness correlations			
Harvesting intensity	-0.083 ± 0.043	-1.939	0.055
Change in harvesting	0.050 ± 0.041	1.223	0.224
Deadwood volume	-0.008 ± 0.040	-0.208	0.835
**Change in deadwood volume**	**-0.073 ± 0.036**	**-2.032**	**0.044**
**Proportion of non-native trees**	**-0.106 ± 0.049**	**-2.172**	**0.032**
**Change in proportion of non-native trees**	**-0.115 ± 0.042**	**-2.711**	**0.008**
Tree diversity	-0.002 ± 0.042	-0.045	0.964
Effective number of layers	-0.043 ± 0.041	-1.049	0.296
Change in effective number of layers	0.040 ± 0.043	0.934	0.352
Canopy openness	-0.015 ± 0.052	-0.285	0.776
**Change in canopy openness**	**-0.130 ± 0.040**	**-3.219**	**0.002**
Forest cover	-0.069 ± 0.048	-1.433	0.154
Disturbance intensity	0.071 ± 0.039	1.822	0.071
**Landscape heterogeneity (PC1)**	**0.124 ± 0.052**	**2.374**	**0.020**
Abundance-accounted richness correlations			
Harvesting intensity	-0.098 ± 0.054	-1.800	0.074
Change in harvesting	0.026 ± 0.052	0.510	0.611
Deadwood volume	0.022 ± 0.050	0.431	0.667
Change in deadwood volume	-0.021 ± 0.045	-0.453	0.651
Proportion of non-native trees	0.094 ± 0.062	1.515	0.132

Change in proportion of non-native trees	-0.047 ± 0.053	-0.880	0.381
Tree diversity	-0.059 ± 0.052	-1.127	0.262
Effective number of layers	-0.057 ± 0.052	-1.110	0.269
Change in effective number of layers	-0.070 ± 0.054	-1.302	0.195
Canopy openness	-0.048 ± 0.066	-0.731	0.466
Change in canopy openness	-0.080 ± 0.049	-1.624	0.107
Forest cover	-0.008 ± 0.057	-0.134	0.894
Disturbance intensity	0.050 ± 0.047	1.057	0.292
Landscape heterogeneity (PC1)	0.068 ± 0.055	1.231	0.221
Abundance correlations			
**Harvesting intensity**	**-0.089 ± 0.044**	**-2.025**	**0.045**
Change in harvesting	0.033 ± 0.042	0.797	0.427
Deadwood volume	-0.030 ± 0.041	-0.735	0.464
Change in deadwood volume	-0.025 ± 0.037	-0.689	0.492
**Proportion of non-native trees**	**-0.177 ± 0.050**	**-3.539**	**0.001**
Change in proportion of non-native trees	-0.074 ± 0.043	-1.708	0.090
**Tree diversity**	**0.103 ± 0.043**	**2.411**	**0.017**
**Effective number of layers**	**-0.084 ± 0.042**	**-2.013**	**0.046**
Change in effective number of layers	0.059 ± 0.043	1.349	0.180
Canopy openness	0.005 ± 0.053	0.087	0.931
**Change in canopy openness**	**-0.104 ± 0.042**	**-2.487**	**0.014**
Forest cover	-0.066 ± 0.050	-1.326	0.187
Disturbance intensity	0.073 ± 0.040	1.832	0.069
Landscape heterogeneity (PC1)	-0.028 ± 0.055	-0.498	0.620
Biomass correlations			
Harvesting intensity	-0.087 ± 0.043	-1.777	0.078
Change in harvesting	0.048 ± 0.040	1.035	0.303
Deadwood volume	0.004 ± 0.040	0.080	0.937
Change in deadwood volume	-0.005 ± 0.036	-0.118	0.906
**Proportion of non-native trees**	**-0.137 ± 0.050**	**-2.451**	**0.016**
**Change in proportion of non-native trees**	**-0.107 ± 0.044**	**-2.207**	**0.029**
Tree diversity	0.071 ± 0.042	1.493	0.138
Effective number of layers	-0.058 ± 0.042	-1.251	0.213
Change in effective number of layers	0.065 ± 0.043	1.340	0.183
Canopy openness	0.021 ± 0.053	0.362	0.718
Change in canopy openness	-0.066 ± 0.042	-1.432	0.155
Forest cover	-0.068 ± 0.077	-1.231	0.221
Disturbance intensity	0.081 ± 0.039	1.805	0.074
Landscape heterogeneity (PC1)	0.075 ± 0.095	1.225	0.223

**Herbivores**			
Richness correlations			
Harvesting intensity	-0.079 ± 0.045	-1.748	0.083
**Change in harvesting**	**0.099 ± 0.043**	**2.329**	**0.022**
Deadwood volume	-0.019 ± 0.042	-0.463	0.644
Change in deadwood volume	-0.065 ± 0.038	-1.738	0.085
Proportion of non-native trees	-0.082 ± 0.051	-1.623	0.107
Change in proportion of non-native trees	-0.049 ± 0.044	-1.103	0.272
Tree diversity	0.034 ± 0.044	0.787	0.433
Effective number of layers	-0.030 ± 0.043	-0.711	0.478
Change in effective number of layers	0.071 ± 0.044	1.605	0.111
Canopy openness	-0.056 ± 0.054	-1.041	0.300
**Change in canopy openness**	**-0.112 ± 0.042**	**-2.676**	**0.009**
Forest cover	-0.073 ± 0.050	-1.472	0.144
Disturbance intensity	0.069 ± 0.040	1.710	0.090
Landscape heterogeneity (PC1)	0.089 ± 0.053	1.687	0.098
Abundance-accounted richness correlations			
Harvesting intensity	-0.035 ± 0.052	-0.673	0.502
Change in harvesting	0.068 ± 0.049	1.371	0.173
Deadwood volume	-0.009 ± 0.048	-0.177	0.859
Change in deadwood volume	-0.006 ± 0.043	-0.129	0.900
Proportion of non-native trees	-0.005 ± 0.059	-0.077	0.939
Change in proportion of non-native trees	0.006 ± 0.051	-0.115	0.908
Tree diversity	-0.032 ± 0.050	-0.627	0.532
Effective number of layers	-0.029 ± 0.049	-0.579	0.564
Change in effective number of layers	0.017 ± 0.051	0.329	0.743
**Canopy openness**	**-0.134 ± 0.062**	**-2.150**	**0.034**
**Change in canopy openness**	**-0.110 ± 0.049**	**-2.260**	**0.026**
Forest cover	-0.078 ± 0.058	-1.340	0.183
Disturbance intensity	0.061 ± 0.047	1.304	0.195
Landscape heterogeneity (PC1)	0.009 ± 0.063	0.141	0.888
Abundance correlations			
**Harvesting intensity**	**-0.123 ± 0.036**	**-3.387**	**0.001**
Change in harvesting	0.023 ± 0.034	0.672	0.503
Deadwood volume	0.054 ± 0.024	1.606	0.111
Change in deadwood volume	-0.021 ± 0.030	-0.676	0.500
**Proportion of non-native trees**	**-0.127 ± 0.041**	**-3.072**	**0.003**
Change in proportion of non-native trees	-0.040 ± 0.036	-1.108	0.270
Tree diversity	0.069 ± 0.035	1.956	0.053
Effective number of layers	-0.045 ± 0.034	-1.307	0.194

Change in effective number of layers	0.055 ± 0.036	1.535	0.127
Canopy openness	0.025 ± 0.043	0.577	0.565
Change in canopy openness	-0.045 ± 0.035	-1.295	0.200
Forest cover	-0.139 ± 0.041	-0.941	0.349
**Disturbance intensity**	**0.083 ± 0.033**	**2.504**	**0.014**
Landscape heterogeneity (PC1)	0.004 ± 0.046	0.096	0.924
Biomass correlations			
Harvesting intensity	-0.093 ± 0.052	-1.780	0.078
Change in harvesting	0.051 ± 0.050	1.031	0.305
Deadwood volume	-0.006 ± 0.049	-0.118	0.906
Change in deadwood volume	0.026 ± 0.044	0.592	0.555
Proportion of non-native trees	0.020 ± 0.060	0.334	0.739
Change in proportion of non-native trees	-0.058 ± 0.052	-1.128	0.261
Tree diversity	0.098 ± 0.051	1.924	0.057
Effective number of layers	-0.042 ± 0.050	-0.837	0.404
Change in effective number of layers	0.099 ± 0.052	1.922	0.057
Canopy openness	-0.012 ± 0.063	-0.191	0.849
Change in canopy openness	-0.054 ± 0.050	-1.085	0.280
Forest cover	0.012 ± 0.059	0.206	0.837
**Disturbance intensity**	**0.095 ± 0.048**	**1.994**	**0.048**
Landscape heterogeneity (PC1)	0.066 ± 0.066	1.005	0.317
**Myceto-detritivores**			
Richness correlations			
Harvesting intensity	-0.074 ± 0.048	-1.556	0.122
Change in harvesting	0.079 ± 0.045	-1.755	0.082
Deadwood volume	-0.011 ± 0.044	-0.243	0.808
Change in deadwood volume	-0.016 ± 0.040	-0.381	0.706
Proportion of non-native trees	-0.070 ± 0.054	-1.293	0.199
**Change in proportion of non-native trees**	**-0.094 ± 0.047**	**-2.004**	**0.047**
Tree diversity	0.007 ± 0.046	0.146	0.884
Effective number of layers	0.021 ± 0.045	0.463	0.644
Change in effective number of layers	0.062 ± 0.047	1.331	0.186
Canopy openness	-0.049 ± 0.057	-0.858	0.397
**Change in canopy openness**	**-0.111 ± 0.044**	**-2.528**	**0.013**
Forest cover	-0.073 ± 0.052	-1.395	0.166
Disturbance intensity	0.051 ± 0.043	1.195	0.234
Landscape heterogeneity (PC1)	0.041 ± 0.054	0.744	0.461
Abundance-accounted richness correlations			
Harvesting intensity	-0.023 ± 0.061	-0.384	0.702

Change in harvesting	0.036 ± 0.058	0.619	0.537
Deadwood volume	0.067 ± 0.056	1.192	0.235
Change in deadwood volume	0.039 ± 0.051	0.762	0.447
Proportion of non-native trees	0.077 ± 0.069	1.115	0.267
Change in proportion of non-native trees	-0.025 ± 0.060	-0.419	0.676
Tree diversity	-0.097 ± 0.059	-1.652	0.101
Effective number of layers	0.001 ± 0.058	0.020	0.984
Change in effective number of layers	-0.040 ± 0.060	-0.667	0.506
Canopy openness	-0.099 ± 0.073	-1.351	0.179
Change in canopy openness	-0.058 ± 0.056	-1.044	0.299
Forest cover	-0.050 ± 0.066	-0.770	0.444
Disturbance intensity	0.045 ± 0.054	0.846	0.400
Landscape heterogeneity (PC1)	0.036 ± 0.066	0.538	0.598
Abundance correlations			
Harvesting intensity	-0.050 ± 0.047	-1.053	0.294
Change in harvesting	0.047 ± 0.045	1.053	0.295
Deadwood volume	-0.059 ± 0.044	-1.332	0.185
Change in deadwood volume	-0.044 ± 0.040	-1.116	0.266
**Proportion of non-native trees**	**-0.121 ± 0.054**	**-2.249**	**0.026**
Change in proportion of non-native trees	-0.055 ± 0.047	-1.185	0.239
Tree diversity	0.071 ± 0.046	1.536	0.127
Effective number of layers	-0.036 ± 0.045	-0.801	0.424
Change in effective number of layers	0.038 ± 0.047	0.802	0.424
Canopy openness	-0.014 ± 0.057	-0.238	0.813
Change in canopy openness	-0.076 ± 0.045	-1.710	0.090
Forest cover	-0.075 ± 0.053	-1.406	0.162
Disturbance intensity	0.040 ± 0.043	0.922	0.358
Landscape heterogeneity (PC1)	-0.012 ± 0.057	-0.208	0.836
Biomass correlations			
Harvesting intensity	-0.023 ± 0.049	-0.456	0.649
Change in harvesting	0.071 ± 0.047	1.514	0.133
Deadwood volume	-0.048 ± 0.046	-1.059	0.292
Change in deadwood volume	0.015 ± 0.041	-0.376	0.708
**Proportion of non-native trees**	**-0.152 ± 0.056**	**-2.708**	**0.008**
**Change in proportion of non-native trees**	**-0.098 ± 0.049**	**-2.012**	**0.046**
**Tree diversity**	**0.118 ± 0.048**	**2.476**	**0.015**
Effective number of layers	-0.005 ± 0.047	-0.103	0.918
Change in effective number of layers	0.081 ± 0.049	1.669	0.098
Canopy openness	0.033 ± 0.059	0.556	0.579
Change in canopy openness	-0.066 ± 0.046	-1.426	0.157

Forest cover	0.022 ± 0.055	0.397	0.692
Disturbance intensity	0.004 ± 0.045	0.089	0.930
Landscape heterogeneity (PC1)	0.049 ± 0.060	0.812	0.419
**Omnivores**			
Richness correlations			
**Harvesting intensity**	**-0.117 ± 0.048**	**-2.463**	**0.015**
Change in harvesting	-0.029 ± 0.045	-0.652	0.515
Deadwood volume	0.004 ± 0.044	0.081	0.935
**Change in deadwood volume**	**-0.087 ± 0.040**	**-2.190**	**0.030**
**Proportion of non-native trees**	**-0.128 ± 0.054**	**-2.369**	**0.019**
Change in proportion of non-native trees	-0.092 ± 0.047	-1.965	0.052
Tree diversity	-0.009 ± 0.046	-0.201	0.841
Effective number of layers	-0.035 ± 0.045	-0.764	0.447
Change in effective number of layers	-0.002 ± 0.047	-0.044	0.965
Canopy openness	0.028 ± 0.057	0.481	0.632
**Change in canopy openness**	**-0.119 ± 0.044**	**2.710**	**0.008**
Forest cover	-0.069 ± 0.052	-1.331	0.187
Disturbance intensity	0.066 ± 0.042	1.564	0.121
Landscape heterogeneity (PC1)	0.068 ± 0.052	1.308	0.201
Abundance-accounted richness correlations			
Harvesting intensity	-0.072 ± 0.060	-1.196	0.234
Change in harvesting	-0.028 ± 0.057	-0.488	0.626
Deadwood volume	-0.006 ± 0.056	-0.114	0.910
Change in deadwood volume	-0.079 ± 0.050	-1.585	0.116
Proportion of non-native trees	-0.068 ± 0.068	-0.991	0.324
Change in proportion of non-native trees	-0.088 ± 0.059	-1.485	0.140
Tree diversity	-0.056 ± 0.058	-0.956	0.341
Effective number of layers	-0.065 ± 0.057	-1.143	0.255
**Change in effective number of layers**	**-0.140 ± 0.059**	**-2.370**	**0.019**
Canopy openness	-0.009 ± 0.072	-0.120	0.905
Change in canopy openness	0.034 ± 0.056	0.605	0.546
Forest cover	0.053 ± 0.066	0.795	0.428
Disturbance intensity	0.050 ± 0.054	0.927	0.356
Landscape heterogeneity (PC1)	0.093 ± 0.071	1.311	0.198
Abundance correlations			
**Harvesting intensity**	**-0.098 ± 0.042**	**-2.334**	**0.021**
Change in harvesting	-0.031 ± 0.040	-0.775	0.440
Deadwood volume	-0.022 ± 0.039	-0.571	0.569
Change in deadwood volume	-0.042 ± 0.035	-1.199	0.233

**Proportion of non-native trees**	**-0.095 ± 0.048**	**-1.990**	**0.049**
**Change in proportion of non-native trees**	**-0.084 ± 0.041**	**-2.028**	**0.045**
Tree diversity	0.039 ± 0.041	0.967	0.336
Effective number of layers	-0.046 ± 0.040	-1.154	0.251
Change in effective number of layers	0.047 ± 0.042	1.132	0.260
Canopy openness	0.015 ± 0.051	0.294	0.769
**Change in canopy openness**	**-0.101 ± 0.039**	**-2.597**	**0.011**
Forest cover	-0.079 ± 0.046	-1.709	0.091
Disturbance intensity	0.057 ± 0.038	1.512	0.134
Landscape heterogeneity (PC1)	0.040 ± 0.048	0.846	0.404
Biomass correlations			
Harvesting intensity	-0.075 ± 0.049	-1.529	0.129
Change in harvesting	0.019 ± 0.046	0.417	0.678
Deadwood volume	0.039 ± 0.045	0.858	0.393
Change in deadwood volume	-0.006 ± 0.041	-0.151	0.881
Proportion of non-native trees	-0.084 ± 0.056	-1.514	0.133
**Change in proportion of non-native trees**	**-0.096 ± 0.048**	**-1.986**	**0.049**
Tree diversity	-0.012 ± 0.047	-0.262	0.794
Effective number of layers	-0.016 ± 0.046	-0.352	0.726
Change in effective number of layers	0.071 ± 0.048	1.472	0.143
Canopy openness	-0.012 ± 0.059	-0.203	0.839
**Change in canopy openness**	**-0.114 ± 0.046**	**-2.470**	**0.015**
**Forest cover**	**-0.189 ± 0.055**	**-3.451**	**0.001**
**Disturbance intensity**	**0.112 ± 0.044**	**2.533**	**0.013**
Landscape heterogeneity (PC1)	0.026 ± 0.059	0.438	0.663
**Carnivores**			
Richness correlations			
Harvesting intensity	-0.061 ± 0.040	-1.530	0.129
Change in harvesting	0.042 ± 0.038	1.105	0.271
Deadwood volume	0.017 ± 0.037	0.470	0.639
Change in deadwood volume	-0.042 ± 0.033	-1.256	0.212
Proportion of non-native trees	-0.052 ± 0.045	-1.142	0.256
Change in proportion of non-native trees	-0.041 ± 0.039	-1.054	0.294
Tree diversity	0.027 ± 0.039	0.698	0.487
Effective number of layers	-0.054 ± 0.038	-1.433	0.154
Change in effective number of layers	0.005 ± 0.039	0.133	0.895
Canopy openness	-0.001 ± 0.048	-0.024	0.981
**Change in canopy openness**	**-0.075 ± 0.038**	**-2.002**	**0.048**
Forest cover	-0.023 ± 0.045	-0.514	0.608
Disturbance intensity	0.035 ± 0.036	0.973	0.333

Landscape heterogeneity (PC1)	0.076 ± 0.049	1.544	0.126
Abundance-accounted richness correlations			
Harvesting intensity	0.003 ± 0.054	0.062	0.951
Change in harvesting	-0.070 ± 0.052	-1.358	0.177
Deadwood volume	0.003 ± 0.051	0.050	0.961
Change in deadwood volume	-0.022 ± 0.045	-0.492	0.623
Proportion of non-native trees	0.078 ± 0.062	1.264	0.209
Change in proportion of non-native trees	-0.039 ± 0.054	-0.731	0.466
Tree diversity	-0.008 ± 0.053	-0.142	0.887
Effective number of layers	-0.074 ± 0.052	-1.430	0.155
Change in effective number of layers	-0.083 ± 0.054	-1.544	0.125
Canopy openness	-0.022 ± 0.066	-0.329	0.743
Change in canopy openness	-0.021 ± 0.052	-0.409	0.684
Forest cover	-0.030 ± 0.061	-0.481	0.631
Disturbance intensity	0.020 ± 0.050	0.403	0.687
Landscape heterogeneity (PC1)	0.124 ± 0.068	1.831	0.070
Abundance correlations			
Harvesting intensity	-0.075 ± 0.043	-1.762	0.081
Change in harvesting	0.048 ± 0.040	1.176	0.242
Deadwood volume	0.026 ± 0.040	0.655	0.514
Change in deadwood volume	-0.027 ± 0.036	-0759	0.449
**Proportion of non-native trees**	**-0.106 ± 0.049**	**-2.189**	**0.031**
Change in proportion of non-native trees	-0.032 ± 0.042	-0.754	0.452
Tree diversity	0.055 ± 0.041	1.334	0.185
Effective number of layers	-0.042 ± 0.041	-1.027	0.306
Change in effective number of layers	0.036 ± 0.042	0.846	0.399
Canopy openness	0.030 ± 0.051	0.575	0.566
Change in canopy openness	-0.074 ± 0.040	-1.834	0.069
Forest cover	-0.002 ± 0.048	-0.033	0.974
Disturbance intensity	0.012 ± 0.039	0.306	0.760
Landscape heterogeneity (PC1)	0.035 ± 0.053	0.668	0.506
Biomass correlations			
Harvesting intensity	0.006 ± 0.051	0.117	0.907
Change in harvesting	0.026 ± 0.048	0.535	0.594
Deadwood volume	-0.016 ± 0.047	-0.344	0.731
Change in deadwood volume	-0.026 ± 0.042	-0.375	0.708
Proportion of non-native trees	-0.100 ± 0.058	-1.730	0.086
Change in proportion of non-native trees	-0.049 ± 0.050	-0.979	0.329
Tree diversity	0.081 ± 0.049	1.643	0.103

Effective number of layers	-0.048 ± 0.048	-0.995	0.322
Change in effective number of layers	0.030 ± 0.050	0.602	0.548
Canopy openness	-0.031 ± 0.061	-0.509	0.611
Change in canopy openness	-0.080 ± 0.048	-1.668	0.098
Forest cover	0.039 ± 0.058	0.677	0.500
Disturbance intensity	0.050 ± 0.046	1.070	0.287
Landscape heterogeneity (PC1)	0.048 ± 0.064	0.749	0.455

Deadwood volume and *Disturbance intensity* were square-root transformed; for *Change in deadwood volume*, *Change in proportion of non-native trees*, *Change in effective number of layers* and *Change in canopy openness* symmetric square-root transformation (separately transforming the absolute values of numbers smaller and larger than zero, subsequently multiplying all transformed values with their original sign) was applied.
