# Supplementary material for: Insect decline in forests depends on species’ traits and may be mitigated by management
Source: Commun Biol. 2023 Apr 4;6:338. doi: 10.1038/s42003-023-04690-9 (PMC10073207; doi:10.1038/s42003-023-04690-9)
Supplement: Supplementary file 5 — Supplementary Data 2 [file 42003_2023_4690_MOESM5_ESM.pdf]

Supplementary Data 2

Insect decline in forests depends on species' traits and can be mitigated by management

Michael Staab, Martin M. Gossner, Nadja K. Simons, Rafael Achury, Didem Ambarlı, Soyeon Bae, Peter Schall, Wolfgang W. Weisser, and Nico Blüthgen

To whom correspondence may be addressed. Email: michael.staab1@tu-darmstadt.de

Results of linear mixed-effects models testing the relationships between site-level correlations with sampling year (Pearson's *r*) of species richness, richness accounted for abundance, abundance and biomass and explanatory variables at the site and landscape scale. Significant relationships ($p < 0.05$) are printed in bold. For explanations of variables see Supplementary Table 1. P-values are calculated based on Kenward-Roger-approximated degrees of freedom. Analyses reported here are identical to those in Supplementary Data 1 but include the respective starting conditions in the first sampling year 2008 as covariate.

	Estimate ± SE	*t*-value	p-value
**Total insect community**			
Richness correlations			
Harvesting intensity	-0.058 ± 0.038	-1.564	0.121
Change in harvesting	0.041 ± 0.035	1.150	0.253
Deadwood volume	0.020 ± 0.035	0.578	0.565
Change in deadwood volume	-0.039 ± 0.032	-1.228	0.222
Proportion of non-native trees	-0.034 ± 0.044	-0.782	0.436
**Change in proportion of non-native trees**	**-0.094 ± 0.037**	**-2.574**	**0.011**
Tree diversity	-0.015 ± 0.036	-0.421	0.675
Effective number of layers	-0.012 ± 0.036	-0.337	0.737
Change in effective number of layers	0.051 ± 0.037	1.394	0.166
Canopy openness	0.014 ± 0.045	0.308	0.760
Change in canopy openness	-0.046 ± 0.036	-1.244	0.216
Forest cover	0.008 ± 0.043	0.179	0.858
Disturbance intensity	0.053 ± 0.033	1.599	0.113
**Landscape heterogeneity (PC1)**	**0.099 ± 0.042**	**2.341**	**0.031**
**Richness in 2008**	**-0.311 ± 0.046**	**-6.713**	**<0.001**
Abundance-accounted richness correlations			
Harvesting intensity	-0.066 ± 0.050	-1.308	0.193

Change in harvesting	0.029 ± 0.047	0.614	0.540
Deadwood volume	0.027 ± 0.046	0.585	0.559
Change in deadwood volume	0.005 ± 0.042	0.117	0.907
Proportion of non-native trees	0.106 ± 0.056	1.891	0.061
Change in proportion of non-native trees	-0.036 ± 0.049	-0.737	0.462
Tree diversity	-0.024 ± 0.048	-0.498	0.619
Effective number of layers	-0.092 ± 0.047	-1.937	0.055
Change in effective number of layers	-0.061 ± 0.049	-1.248	0.215
Canopy openness	-0.080 ± 0.060	-1.335	0.184
Change in canopy openness	-0.055 ± 0.045	-1.217	0.226
Forest cover	0.037 ± 0.053	0.690	0.492
Disturbance intensity	0.077 ± 0.043	1.783	0.077
Landscape heterogeneity (PC1)	0.087 ± 0.051	1.721	0.088
**Abundance-accounted richness in 2008**	**-0.210 ± 0.041**	**-5.096**	**<0.001**
Abundance correlations			
**Harvesting intensity**	**-0.076 ± 0.038**	**-1.986**	**0.049**
Change in harvesting	0.018 ± 0.036	0.486	0.628
Deadwood volume	-0.007 ± 0.035	-0.194	0.847
Change in deadwood volume	-0.009 ± 0.032	-0.266	0.791
**Proportion of non-native trees**	**-0.110 ± 0.045**	**-2.455**	**0.016**
Change in proportion of non-native trees	-0.059 ± 0.038	-1.582	0.116
Tree diversity	0.057 ± 0.038	1.515	0.133
Effective number of layers	-0.019 ± 0.037	-0.513	0.609
Change in effective number of layers	0.062 ± 0.038	1.635	0.105
Canopy openness	0.060 ± 0.047	1.279	0.203
Change in canopy openness	-0.006 ± 0.038	-0.151	0.880
Forest cover	0.006 ± 0.043	0.134	0.894
Disturbance intensity	0.010 ± 0.034	0.299	0.766
Landscape heterogeneity (PC1)	-0.016 ± 0.043	-0.361	0.722
**Abundance in 2008**	**-0.353 ± 0.047**	**-7.445**	**<0.001**
Biomass correlations			
Harvesting intensity	-0.040 ± 0.043	-0.915	0.362
Change in harvesting	0.051 ± 0.040	1.267	0.208
Deadwood volume	0.024 ± 0.040	0.594	0.553
Change in deadwood volume	0.029 ± 0.036	0.804	0.423
Proportion of non-native trees	-0.062 ± 0.050	-1.239	0.220
Change in proportion of non-native trees	-0.080 ± 0.042	-1.823	0.071
Tree diversity	0.030 ± 0.042	0.715	0.476
Effective number of layers	0.012 ± 0.042	0.275	0.784
**Change in effective number of layer**s	**0.094 ± 0.042**	**2.216**	**0.029**

Canopy openness	0.051 ± 0.051	0.982	0.328
Change in canopy openness	-0.008 ± 0.041	-0.182	0.866
Forest cover	0.027 ± 0.050	0.545	0.590
Disturbance intensity	0.029 ± 0.039	0.729	0.468
Landscape heterogeneity (PC1)	0.030 ± 0.052	0.578	0.566
**Biomass in 2008**	**-0.394 ± 0.061**	**-6.478**	**<0.001**
**Herbivores**			
Richness correlations			
**Harvesting intensity**	**-0.098 ± 0.038**	**-2.593**	**0.011**
Change in harvesting	0.040 ± 0.037	1.105	0.271
Deadwood volume	0.008 ± 0.035	0.232	0.817
Change in deadwood volume	-0.036 ± 0.032	-1.130	0.261
**Proportion of non-native trees**	**-0.093 ± 0.043**	**-2.168**	**0.032**
Change in proportion of non-native trees	-0.053 ± 0.037	-1.435	0.154
Tree diversity	0.051 ± 0.037	1.396	0.165
Effective number of layers	-0.027 ± 0.036	-0.751	0.454
Change in effective number of layers	0.045 ± 0.037	1.213	0.228
Canopy openness	0.048 ± 0.048	0.999	0.320
Change in canopy openness	-0.012 ± 0.038	-0.318	0.751
Forest cover	-0.026 ± 0.042	-0.630	0.530
Disturbance intensity	0.046 ± 0.034	1.345	0.182
**Landscape heterogeneity (PC1)**	**0.093 ± 0.043**	**2.141**	**0.042**
**Richness in 2008**	**-0.242 ± 0.033**	**-7.312**	**<0.001**
Abundance-accounted richness correlations			
Harvesting intensity	-0.058 ± 0.049	-1.180	0.240
Change in harvesting	0.029 ± 0.047	0.620	0.536
Deadwood volume	0.019 ± 0.046	0.412	0.681
Change in deadwood volume	0.006 ± 0.041	0.147	0.884
Proportion of non-native trees	0.010 ± 0.055	0.172	0.864
Change in proportion of non-native trees	0.002 ± 0.048	0.039	0.969
Tree diversity	-0.011 ± 0.048	-0.239	0.811
Effective number of layers	-0.041 ± 0.046	-0.887	0.377
Change in effective number of layers	0.006 ± 0.048	0.134	0.894
Canopy openness	-0.079 ± 0.060	-1.309	0.193
Change in canopy openness	-0.058 ± 0.048	-1.209	0.229
Forest cover	-0.076 ± 0.055	-1.394	0.166
Disturbance intensity	0.055 ± 0.044	1.237	0.219
Landscape heterogeneity (PC1)	0.015 ± 0.062	0.243	0.809
**Abundance-accounted richness in 2008**	**-0.168 ± 0.040**	**-4.189**	**<0.001**

Abundance correlations			
**Harvesting intensity**	**-0.107 ± 0.032**	**-3.343**	**0.001**
Change in harvesting	0.001 ± 0.031	0.021	0.987
Deadwood volume	0.053 ± 0.030	1.788	0.076
Change in deadwood volume	-0.001 ± 0.027	-0.020	0.984
**Proportion of non-native trees**	**-0.167 ± 0.037**	**-4.521**	**<0.001**
Change in proportion of non-native trees	-0.031 ± 0.032	-0.995	0.322
**Tree diversity**	**0.067 ± 0.031**	**2.158**	**0.033**
Effective number of layers	-0.012 ± 0.031	-0.371	0.711
Change in effective number of layers	0.040 ± 0.032	1.274	0.205
**Canopy openness**	**0.086 ± 0.040**	**2.163**	**0.033**
Change in canopy openness	0.001 ± 0.031	0.019	0.985
Forest cover	0.004 ± 0.037	0.111	0.912
Disturbance intensity	0.055 ± 0.030	1.842	0.068
Landscape heterogeneity (PC1)	-0.010 ± 0.041	-0.248	0.805
**Abundance in 2008**	**-0.200 ± 0.033**	**-6.121**	**<0.001**
Biomass correlations			
Harvesting intensity	-0.030 ± 0.044	-0.677	0.500
Change in harvesting	0.032 ± 0.041	0.761	0.448
Deadwood volume	-0.016 ± 0.040	-0.387	0.700
Change in deadwood volume	0.067 ± 0.037	1.820	0.071
Proportion of non-native trees	-0.017 ± 0.050	-0.346	0.730
Change in proportion of non-native trees	-0.007 ± 0.043	-0.159	0.874
Tree diversity	0.076 ± 0.042	1.791	0.076
Effective number of layers	0.017 ± 0.041	0.423	0.673
**Change in effective number of layers**	**0.113 ± 0.043**	**2.638**	**0.009**
Canopy openness	0.034 ± 0.053	0.647	0.519
Change in canopy openness	-0.007 ± 0.040	-0.173	0.863
**Forest cover**	**0.107 ± 0.050**	**2.177**	**0.031**
Disturbance intensity	0.064 ± 0.038	1.692	0.093
Landscape heterogeneity (PC1)	0.054 ± 0.045	1.208	0.229
**Biomass in 2008**	**-0.390 ± 0.040**	**-9.730**	**<0.001**
**Myceto-detritivores**			
Richness correlations			
Harvesting intensity	-0.031 ± 0.041	-0.746	0.457
**Change in harvesting**	**0.078 ± 0.039**	**2.014**	**0.046**
Deadwood volume	0.010 ± 0.038	0.267	0.790
Change in deadwood volume	0.012 ± 0.034	0.351	0.726
Proportion of non-native trees	0.002 ± 0.048	0.040	0.968
Change in proportion of non-native trees	-0.055 ± 0.041	-1.359	0.178

Tree diversity	0.002 ± 0.040	0.040	0.968
Effective number of layers	0.073 ± 0.040	1.834	0.069
**Change in effective number of layers**	**0.091 ± 0.041**	**2.241**	**0.027**
Canopy openness	-0.030 ± 0.050	-0.620	0.537
Change in canopy openness	-0.050 ± 0.040	-1.269	0.207
Forest cover	0.005 ± 0.047	0.115	0.909
Disturbance intensity	0.031 ± 0.037	0.832	0.407
Landscape heterogeneity (PC1)	-0.015 ± 0.050	-0.302	0.764
**Richness in 2008**	**-0.328 ± 0.049**	**-6.681**	**<0.001**
Abundance-accounted richness correlations			
Harvesting intensity	0.055 ± 0.055	0.987	0.326
Change in harvesting	0.062 ± 0.051	1.209	0.229
Deadwood volume	0.041 ± 0.050	0.815	0.417
Change in deadwood volume	0.074 ± 0.045	1.632	0.105
Proportion of non-native trees	0.090 ± 0.061	1.475	0.143
Change in proportion of non-native trees	0.009 ± 0.053	0.173	0.863
Tree diversity	-0.039 ± 0.053	-0.742	0.459
Effective number of layers	-0.003 ± 0.051	-0.052	0.959
Change in effective number of layers	-0.035 ± 0.053	-0.653	0.515
**Canopy openness**	**-0.144 ± 0.065**	**-2.202**	**0.030**
Change in canopy openness	-0.053 ± 0.049	-1.089	0.278
Forest cover	0.028 ± 0.059	0.476	0.635
Disturbance intensity	0.083 ± 0.047	1.770	0.079
Landscape heterogeneity (PC1)	-0.051 ± 0.056	-0.897	0.371
**Abundance-accounted richness in 2008**	**-0.257 ± 0.043**	**-5.987**	**<0.001**
Abundance correlations			
Harvesting intensity	-0.059 ± 0.043	-1.095	0.172
Change in harvesting	0.030 ± 0.041	0.735	0.464
Deadwood volume	-0.023 ± 0.040	-0.570	0.570
Change in deadwood volume	-0.038 ± 0.036	-1.067	0.288
Proportion of non-native trees	-0.059 ± 0.050	-1.173	0.243
Change in proportion of non-native trees	-0.042 ± 0.042	-0.986	0.326
Tree diversity	0.029 ± 0.042	0.695	0.488
Effective number of layers	0.013 ± 0.042	0.309	0.758
Change in effective number of layers	0.060 ± 0.043	1.417	0.159
Canopy openness	0.033 ± 0.052	0.627	0.532
Change in canopy openness	-0.021 ± 0.042	-0.506	0.614
Forest cover	-0.047 ± 0.048	-0.965	0.337
Disturbance intensity	0.004 ± 0.039	0.103	0.918
Landscape heterogeneity (PC1)	-0.022 ± 0.053	-0.413	0.681

**Abundance in 2008**	**-0.266 ± 0.050**	**-5.222**	**<0.001**
Biomass correlations			
Harvesting intensity	0.001 ± 0.040	0.025	0.980
Change in harvesting	0.071 ± 0.038	1.879	0.063
Deadwood volume	-0.044 ± 0.037	-1.206	0.230
Change in deadwood volume	0.012 ± 0.033	0.359	0.721
Proportion of non-native trees	-0.077 ± 0.046	-1.665	0.098
**Change in proportion of non-native trees**	**-0.080 ± 0.039**	**-2.048**	**0.043**
**Tree diversity**	**0.079 ± 0.039**	**2.043**	**0.033**
Effective number of layers	0.055 ± 0.038	1.436	0.154
**Change in effective number of layers**	**0.113 ± 0.039**	**2.872**	**0.005**
Canopy openness	0.061 ± 0.048	1.277	0.204
Change in canopy openness	-0.035 ± 0.037	-0.954	0.343
Forest cover	0.075 ± 0.044	1.711	0.090
Disturbance intensity	-0.024 ± 0.035	-0.692	0.491
Landscape heterogeneity (PC1)	-0.008 ± 0.044	-0.174	0.865
**Biomass in 2008**	**-0.354 ± 0.041**	**-8.591**	**<0.001**
**Omnivores**			
Richness correlations			
**Harvesting intensity**	**-0.082 ± 0.041**	**-1.993**	**0.049**
Change in harvesting	-0.026 ± 0.039	-0.667	0.506
Deadwood volume	0.043 ± 0.038	1.128	0.262
Change in deadwood volume	-0.042 ± 0.035	-1.229	0.222
Proportion of non-native trees	-0.033 ± 0.048	-0.680	0.500
Change in proportion of non-native trees	-0.059 ± 0.040	-1.458	0.148
Tree diversity	-0.015 ± 0.040	-0.386	0.700
Effective number of layers	0.004 ± 0.039	0.108	0.914
Change in effective number of layers	0.005 ± 0.040	0.135	0.893
Canopy openness	0.051 ± 0.049	1.047	0.297
Change in canopy openness	-0.022 ± 0.041	-0.533	0.595
Forest cover	0.022 ± 0.047	0.471	0.638
Disturbance intensity	0.039 ± 0.037	1.053	0.295
Landscape heterogeneity (PC1)	0.031 ± 0.050	0.613	0.542
**Richness in 2008**	**-0.345 ± 0.051**	**-6.768**	**<0.001**
Abundance-accounted richness correlations			
Harvesting intensity	-0.047 ± 0.055	-0.858	0.393
Change in harvesting	-0.017 ± 0.052	-0.319	0.750
Deadwood volume	0.034 ± 0.051	0.677	0.500
Change in deadwood volume	-0.048 ± 0.046	-1.036	0.302

Proportion of non-native trees	-0.052 ± 0.062	-0.836	0.405
Change in proportion of non-native trees	-0.061 ± 0.054	-1.130	0.261
Tree diversity	-0.010 ± 0.053	-0.192	0.848
Effective number of layers	-0.042 ± 0.052	-0.808	0.421
**Change in effective number of layers**	**-0.114 ± 0.054**	**-2.116**	**0.036**
Canopy openness	-0.020 ± 0.066	-0.305	0.761
Change in canopy openness	0.049 ± 0.050	0.979	0.330
Forest cover	0.055 ± 0.058	0.945	0.349
Disturbance intensity	0.063 ± 0.048	1.321	0.191
Landscape heterogeneity (PC1)	0.062 ± 0.057	1.098	0.293
**Abundance-accounted richness in 2008**	**-0.235 ± 0.044**	**-5.330**	**<0.001**
Abundance correlations			
**Harvesting intensity**	**-0.083 ± 0.039**	**-2.126**	**0.036**
Change in harvesting	-0.032 ± 0.037	-0.876	0.383
Deadwood volume	-0.002 ± 0.036	-0.060	0.952
Change in deadwood volume	-0.024 ± 0.033	-0.732	0.466
Proportion of non-native trees	-0.023 ± 0.047	-0.496	0.621
Change in proportion of non-native trees	-0.062 ± 0.039	-1.597	0.113
Tree diversity	0.018 ± 0.038	0.480	0.632
Effective number of layers	-0.019 ± 0.038	-0.517	0.606
Change in effective number of layers	0.045 ± 0.039	1.159	0.249
Canopy openness	0.043 ± 0.047	0.906	0.367
Change in canopy openness	-0.045 ± 0.039	-1.139	0.259
Forest cover	-0.031 ± 0.045	-0.685	0.495
Disturbance intensity	0.030 ± 0.036	0.825	0.411
Landscape heterogeneity (PC1)	-0.008 ± 0.049	-0.156	0.876
**Abundance in 2008**	**-0.256 ± 0.057**	**-4.523**	**<0.001**
Biomass correlations			
Harvesting intensity	-0.057 ± 0.046	-1.238	0.218
Change in harvesting	0.017 ± 0.043	0.394	0.694
Deadwood volume	0.077 ± 0.043	1.774	0.079
Change in deadwood volume	0.012 ± 0.038	0.304	0.762
Proportion of non-native trees	-0.004 ± 0.055	-0.078	0.938
**Change in proportion of non-native trees**	**-0.092 ± 0.045**	**-2.042**	**0.043**
Tree diversity	-0.048 ± 0.045	-1.061	0.291
Effective number of layers	0.008 ± 0.044	0.191	0.849
Change in effective number of layers	0.077 ± 0.044	1.719	0.088
Canopy openness	0.006 ± 0.055	0.114	0.910
Change in canopy openness	-0.065 ± 0.045	-1.446	0.151
**Forest cover**	**-0.122 ± 0.054**	**-2.271**	**0.029**

Disturbance intensity	0.0705± 0.043	1.761	0.081
Landscape heterogeneity (PC1)	-0.028 ± 0.058	-0.480	0.632
**Biomass in 2008**	**-0.264 ± 0.061**	**-4.344**	**<0.001**
**Carnivores**			
Richness correlations			
Harvesting intensity	-0.064 ± 0.037	-1.704	0.091
Change in harvesting	0.042 ± 0.035	1.178	0.241
Deadwood volume	0.046 ± 0.034	1.328	0.189
Change in deadwood volume	-0.020 ± 0.031	-0.637	0.525
Proportion of non-native trees	0.005 ± 0.044	0.108	0.914
Change in proportion of non-native trees	-0.042 ± 0.037	-1.154	0.251
Tree diversity	0.005 ± 0.037	0.144	0.886
Effective number of layers	-0.048 ± 0.035	-1.364	0.175
Change in effective number of layers	0.017 ± 0.037	0.474	0.637
Canopy openness	-0.002 ± 0.045	-0.037	0.971
Change in canopy openness	-0.042 ± 0.036	-1.167	0.246
Forest cover	-0.005 ± 0.042	-0.125	0.901
Disturbance intensity	0.040 ± 0.032	1.247	0.215
Landscape heterogeneity (PC1)	0.048 ± 0.038	1.261	0.210
**Richness in 2008**	**-0.229 ± 0.038**	**-6.028**	**<0.001**
Abundance-accounted richness correlations			
Harvesting intensity	-0.032 ± 0.051	-0.625	0.533
Change in harvesting	-0.064 ± 0.048	-1.327	0.189
Deadwood volume	0.008 ± 0.047	0.170	0.866
Change in deadwood volume	-0.017 ± 0.042	-0.399	0.691
Proportion of non-native trees	0.095 ± 0.058	1.644	0.103
Change in proportion of non-native trees	-0.040 ± 0.050	-0.811	0.419
Tree diversity	0.018 ± 0.049	0.355	0.723
**Effective number of layers**	**-0.102 ± 0.048**	**-2.101**	**0.038**
Change in effective number of layers	-0.067 ± 0.050	-1.336	0.184
Canopy openness	-0.027 ± 0.061	-0.436	0.664
Change in canopy openness	-0.057 ± 0.048	-1.185	0.239
Forest cover	-0.038 ± 0.057	-0.675	0.501
Disturbance intensity	0.033 ± 0.046	0.712	0.478
**Landscape heterogeneity (PC1)**	**0.147 ± 0.063**	**2.341**	**0.021**
**Abundance-accounted richness in 2008**	**-0.195 ± 0.042**	**-4.602**	**<0.001**
Abundance correlations			
Harvesting intensity	-0.053 ± 0.038	-1.384	0.169
Change in harvesting	0.042 ± 0.036	1.147	0.254

Deadwood volume	0.049 ± 0.035	1.386	0.168
Change in deadwood volume	-0.008 ± 0.032	-0.234	0.815
Proportion of non-native trees	-0.056 ± 0.044	-1.264	0.209
Change in proportion of non-native trees	-0.036 ± 0.037	-0.960	0.339
Tree diversity	0.010 ± 0.038	0.270	0.788
Effective number of layers	-0.018 ± 0.036	-0.498	0.619
Change in effective number of layers	0.035 ± 0.038	0.932	0.353
Canopy openness	0.032 ± 0.046	0.691	0.491
Change in canopy openness	-0.002 ± 0.037	-0.062	0.951
Forest cover	0.037 ± 0.042	0.878	0.382
Disturbance intensity	0.001 ± 0.033	0.024	0.981
Landscape heterogeneity (PC1)	0.019 ± 0.039	0.485	0.628
**Abundance in 2008**	**-0.266 ± 0.038**	**-7.098**	**<0.001**
Biomass correlations			
Harvesting intensity	0.035 ± 0.044	0.801	0.425
Change in harvesting	0.059 ± 0.042	1.406	0.162
Deadwood volume	0.008 ± 0.041	0.192	0.848
Change in deadwood volume	0.025 ± 0.037	0.669	0.505
Proportion of non-native trees	-0.020 ± 0.051	-0.383	0.703
Change in proportion of non-native trees	0.003 ± 0.044	0.073	0.942
Tree diversity	0.026 ± 0.043	0.601	0.549
Effective number of layers	-0.028 ± 0.042	-0.678	0.499
Change in effective number of layers	0.049 ± 0.043	1.130	0.261
Canopy openness	-0.035 ± 0.053	-0.653	0.515
Change in canopy openness	-0.033 ± 0.042	-0.791	0.430
Forest cover	0.081 ± 0.050	1.628	0.106
Disturbance intensity	0.029 ± 0.040	0.736	0.463
Landscape heterogeneity (PC1)	0.012 ± 0.055	0.217	0.829
**Biomass in 2008**	**-0.306 ± 0.046**	**-6.626**	**<0.001**

Deadwood volume and *Disturbance intensity* were square-root transformed; for *Change in deadwood volume*, *Change in proportion of non-native trees*, *Change in effective number of layers* and *Change in canopy openness* symmetric square-root transformation (separately transforming the absolute values of numbers smaller and larger than zero, subsequently multiplying all transformed values with their original sign) was applied; conditions in the first year (*values in 2008*) were log-transformed.
